# Acute Exercise and Appetite-Regulating Hormones in Overweight and Obese Individuals: A Meta-Analysis

**DOI:** 10.1155/2016/2643625

**Published:** 2016-12-27

**Authors:** Jessica Anne Douglas, Kevin Deighton, Jan Maria Atkinson, Vahid Sari-Sarraf, David John Stensel, Greg Atkinson

**Affiliations:** ^1^School of Sport, Exercise and Health Sciences, Loughborough University, Loughborough, UK; ^2^The Leicester-Loughborough Diet, Lifestyle & Physical Activity Biomedical Research Unit, University Hospitals of Leicester and Loughborough University, Tees Valley, UK; ^3^Institute for Sport, Physical Activity & Leisure, Leeds Beckett University, Leeds, UK; ^4^Health and Social Care Institute, Teesside University, Middlesbrough, UK; ^5^Department of Exercise Physiology, Faculty of Physical Education and Sport Sciences, University of Tabriz, Tabriz, Iran

## Abstract

In lean individuals, acute aerobic exercise is reported to transiently suppress sensations of appetite, suppress blood concentrations of acylated ghrelin (AG), and increase glucagon-like peptide-1 (GLP-1) and peptide-YY (PYY). Findings in overweight/obese individuals have yet to be synthesised. In this systematic review and meta-analysis, we quantified the effects that acute exercise has on AG and total PYY and GLP-1 in overweight/obese individuals. The potential for body mass index (BMI) to act as a moderator for AG was also explored. Six published studies (73 participants, 78% male, mean BMI: 30.6 kg·m^−2^) met the inclusion criteria. Standardised mean differences (SMDs) and standard errors were extracted for AG and total PYY and GLP-1 concentrations in control and exercise trials and synthesised using a random effects meta-analysis model. BMI was the predictor in metaregression for AG. Exercise moderately suppressed AG area-under-the-curve concentrations (pooled SMD: −0.34, 95% CI: −0.53 to −0.15). The magnitude of this reduction was greater for higher mean BMIs (pooled metaregression slope: −0.04 SMD/kg·m^−2^ (95% CI: −0.07 to 0.00)). Trivial SMDs were obtained for total PYY (0.10, 95% CI: −0.13 to 0.31) and GLP-1 (−0.03, 95% CI: −0.18 to 0.13). This indicates that exercise in overweight/obese individuals moderately alters AG in a direction that could be associated with decreased hunger and energy intake. This trial is registered with PROSPERO: CRD42014006265.

## 1. Introduction

Adults with a body mass index (BMI) of equal to or greater than 25 kg·m^−2^ are classified as overweight, whilst those with a BMI equal to or greater than 30 kg·m^−2^ are classified as obese [1]. In 2008, it was estimated that 1.4 billion adults were overweight worldwide. Of these, 200 million men and nearly 300 million women were obese [1]. Obesity is associated with an increased risk of type 2 diabetes, coronary heart disease, and some types of cancer, as well as strokes [2]. The health risks and financial burden associated with overweight and obesity are causes for concern. Governments are developing schemes and guidelines to help counter overweight and obesity. Alongside limiting energy intake, individuals are advised to take part in regular physical activity. In the UK, healthy adults are advised to perform at least 150 min of moderate intensity aerobic activity per week and to combine this with muscle strengthening exercise twice a week [3].

*“Exercise-induced anorexia”* was a term coined in 1994 by King and colleagues to describe the condition where appetite is suppressed after acute exercise [4]. King et al. [4] showed that appetite was temporarily suppressed during and after high intensity exercise in lean healthy males. Subsequent researchers confirmed these earlier findings [5–9]. At rest, feelings of hunger are mediated by gut hormones such as acylated ghrelin, peptide-YY (PYY), and glucagon-like peptide-1 (GLP-1) [10–12]. It has been hypothesised that “exercise-induced anorexia” is mediated by altered concentrations of these hormones. For example, acylated ghrelin (an appetite-stimulating hormone) has been found to be suppressed after vigorous exercise [6, 9, 13]. In contrast, circulating concentrations of PYY and GLP-1 (satiety hormones) have been shown to increase after exercise in healthy lean adults [6, 9, 13]. Researchers have also examined the effects that exercise has on energy intake after exercise. The majority of studies indicate that individuals do not compensate for the energy expended during exercise in the immediate hours after exercise [14]. Therefore, these individuals are in an energy deficit, and if maintained over time this could result in weight loss.

Most studies on exercise and appetite regulation involve crossover designs and relatively small sample sizes. Meta-analyses can be useful to quantify the effects of an intervention with greater precision from a pooled estimate. A standardised mean difference (SMD) is often reported. Recently, the effects of acute exercise on appetite regulatory hormones were examined in lean and overweight/obese individuals [15]. It was concluded that an acute bout of exercise suppresses acylated ghrelin (SMD: 0.20) and increases PYY (SMD: 0.24), GLP-1 (SMD: 0.28), and pancreatic polypeptide (SMD: 0.50). Of the 25 studies included in this review, only two involved overweight/obese individuals. Clearly, there are fewer studies on overweight/obese individuals and no previous systematic review or meta-analysis has been undertaken in this population. Such a review would capture new studies involving overweight/obese individuals and clarify whether they respond in a similar way to their lean counterparts. This in turn could enhance the understanding of the role that exercise plays in weight maintenance and control. Therefore, we aimed to synthesise this evidence from studies investigating acute exercise bouts and circulating concentrations of acylated ghrelin, total PYY, and total GLP-1, measured in overweight/obese participants.

## 2. Methods

### 2.1. Data Source

A systematic review of peer-reviewed studies was undertaken comparing concentrations of appetite regulatory hormones, quantified as an area under the curve (AUC). The review was registered with the PROSPERO database (CRD42014006265).

The literature search was conducted by an information specialist (JA) using commonly used research databases (Applied Social Sciences Index and Abstracts (ASSIA), Campbell Collaboration, Centre for Review and Dissemination, Database of Promoting Health Effectiveness Reviews (DoPHER), Cochrane Central Register of Controlled Trials (CENTRAL), Cochrane Database of Systematic Reviews, Cochrane Methodology Register, Database of Abstracts of Reviews or Effects (DARE), EMBASE, NHS Economic Evaluation Database (NHS EED), PROSPERO, PubMed, PsycINFO MEDLINE (Ovid), Sports Discus, Scopus, Web of Knowledge, and CINAHL). These databases were searched in January 2014 with an update search in June 2014 and October 2016. Keyword searches were performed for “exercise”, “physical activity”, “energy expenditure”, “energy intake”, “appetite”, “hunger”, “food intake”, “ghrelin”, “acylated ghrelin”, “total ghrelin”, “acyl ghrelin”, “peptide YY”, “PYY”, “peptide YY_3-36_”, “PYY_3-36_”, “total PYY”, “glucagon-like peptide-1”, “GLP-1”, “active GLP-1”, “GLP-1(7-36)”, “GLP-1(9-36)”, “obese”, “overweight”, and “appetite hormones”. Details of the search strategy are provided in the Supplementary Material available at http://dx.doi.org/10.1155/2016/2643625.

### 2.2. Inclusion Criteria

For inclusion, studies were required to meet the following criteria: participants in the study were overweight/obese adults, with no history of diabetes or gastrointestinal, inflammatory, metabolic, cardiovascular, or psychological disease; in addition, participants were required to be nonsmokers. Study selection criteria were not limited by the duration or observation period after exercise. To maximise search sensitivity, there were also no limitations on the intensity, duration, or modality of the acute exercise bout.

All studies were required to have a control condition which was completed by the same participants who completed the exercise condition. The control condition was required to be identical to that of the exercise condition, minus the exercise bout.

Since the interventions were exercise bouts, investigators were not blinded. Studies were included if they were published in peer-reviewed journals or were available in conference proceedings, theses, or dissertations. A broad range of sources for study inclusion were chosen to minimise the risk of small study effects, which can occur if only published studies are included.

### 2.3. Exclusion Criteria

Studies were excluded if they did not measure acylated ghrelin, total PYY, or total GLP-1 responses to an exercise bout in overweight/obese individuals. Studies were also excluded if they did not include a control trial.

### 2.4. Study Selection

**Figure 1:**
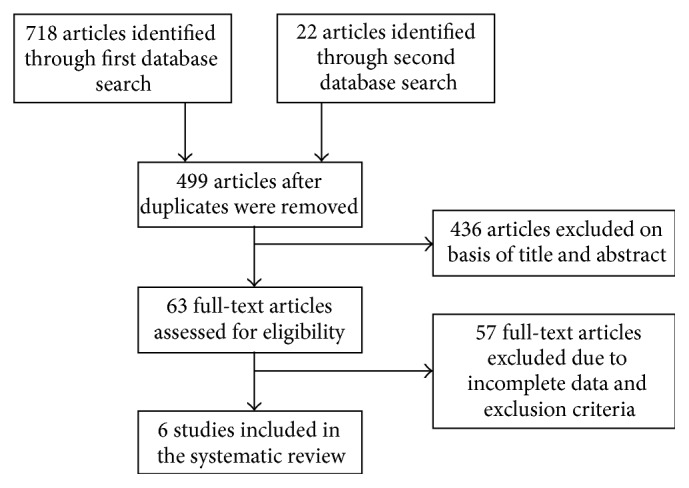
Flowchart of study selection.

Two members of the research team (JAD and KD) independently selected the studies for inclusion in the meta-analysis and later compared notes to reach a mutual consensus. Potential studies were identified by examining the abstracts and full-text copies were obtained if they met the initial criteria of evaluating appetite hormone changes in response to an acute exercise bout. In the original literature search conducted in January 2014, five studies met the inclusion criteria. Two update searches, conducted in June 2014 and October 2016, identified one further study. Together, six studies met the inclusion criteria for the current meta-analysis (Figure 1).

### 2.5. Data Synthesis

Included studies were assessed for quality and validity independently by two authors (JAD and KD), using established criteria (Physiotherapy Evidence Database [PEDro], http://www.pedro.org.au/english/downloads/pedro%20scale/). Data on the study methods, sample size, participant characteristics, blood analytical methods, exercise intervention information, and hormone (pmol·L^−1^ h^−1^, pg·mL^−1^ h^−1^, and *μ*U·mL^−1^ h^−1^) and appetite AUC data were extracted for both control and exercise conditions by one author (JAD) into a computerised spreadsheet. Data entry was checked by one other author (KD), and discrepancies were discussed and checked again. If standard error of the mean (SEM) was reported, it was converted to standard deviations [16].

### 2.6. Meta-Analysis Procedures

Comprehensive meta-analysis software (Version 2.2.064; Biostat, Englewood, NJ, USA) was used to conduct a random effects (DerSimonian-Laird inverse variance approach) meta-analysis of the mean difference in acylated ghrelin, total PYY, and total GLP-1 during control and exercise trials [16]. The inputted data included sample sizes, AUCs for the control and exercise conditions (with their respective standard deviations), and an imputed correlation coefficient to take into account the fact that all studies were crossover in nature. These correlation coefficients were estimated from prior reliability studies in our laboratory and were as follows: acylated ghrelin *r* = 0.93, total PYY *r* = 0.71, and GLP-1 *r* = 0.94. The software calculated the pooled standardised difference in means to determine the effect size [17]. All data are presented as means (95% confidence interval).

We interpreted standardised mean difference (SMD) values of <0.2 as trivial, 0.2–0.3 as small, 0.4–0.8 as moderate, and >0.8 as large [18]. A negative effect size indicates that exercise was associated with decreased hormone concentrations, whilst a positive effect size indicates that hormone concentrations increased with exercise [19]. Heterogeneity was explored using a *Q*-test, *I*-square statistic, and the tau-squared statistic.

### 2.7. Metaregression Analyses for BMI of Acylated Ghrelin AUC between Exercise and Control Conditions

BMI was used as a moderator in a metaregression analysis (methods-of-moments model), to determine whether BMI could explain the variation in effect size values seen between studies for acylated ghrelin concentrations [17]. Mean BMIs were pooled from studies collected in the current review together with those reported for lean individuals in a recent review by Schubert et al. [15]. Mean BMI was included as a moderating variable, as a negative association exists between study mean BMI and acylated ghrelin concentrations [25]. This analysis was only performed for acylated ghrelin as there were not enough studies (*N* < 3) reporting data for total PYY or total GLP-1 to obtain sufficiently precise and meaningful estimations of metaregression slope.

### 2.8. Exploration of Small Study Effects

Small study effects were explored with a funnel plot of standard difference in means versus associated standard errors [26] and by quantifying Egger's linear regression intercept. A large and statistically significant Egger statistic indicates the presence of a small study effect. This analysis was only performed for acylated ghrelin as there were not enough studies reporting data for a precise exploration of total PYY and total GLP-1.

## 3. Results

### 3.1. Overview

Six studies involving a total of 73 participants met the inclusion criteria for the meta-analysis. All of these had been published (or accepted for publication) in peer-reviewed scientific journals. The experimental trials in each study lasted between 2 and 3 h, with exercise conducted in a fasting condition or following a standardised breakfast. Three studies included standardised meals before the exercise bout [13, 21, 24] and four studies included ad libitum meals after the exercise bout [13, 21, 22, 24]. Blood samples were collected at regular intervals throughout all trials.

The included studies are summarised in Table 1. The majority of studies recruited participants of the same sex. One study recruited both males and females [21]. Two of the studies involved more than one exercise intensity trial [21, 22] and are reported in the analysis as “multiple trials.” Accounting for these, the total number of trials is 10, each including one control and one exercise condition. Six studies (10 trials) reported acylated ghrelin AUC data, two studies (four trials) reported total PYY AUC data, and two studies (four trials) reported total GLP-1 AUC data. Of the 10 trials, one used treadmill walking as the mode of exercise, two used treadmill running, and seven used a cycle ergometer. The mean PEDro score for the six studies was 6 ± 0, rating all studies to have “good” methodological quality.

### 3.2. Participant Demographics and Exercise Intervention Characteristics

Of the 73 participants included in the meta-analysis, 57 were men (78%) and 16 were women (22%). BMI values of the 73 participants ranged from 27.7 to 32.7 kg·m^−2^ (mean 30.6 kg·m^−2^). Four studies used exercise which was aerobic in nature [13, 22–24], and two compared aerobic exercise with two variations of high intensity exercise [21, 22]. The exercise interventions lasted between 9 and 60 min (mean 34 min), and exercise intensity was set between 50 and 65% VO_2_ peak (mean 58% VO_2_ peak, *N* = 6) or 72.5 and 87.5%  HR_max_ (mean 79.4%  HR_max_, *N* = 4). Between 7 and 19 participants took part in each study (mean 12 participants) (see Table 1 for summaries of study protocols).

### 3.3. Meta-Analysis

Individual study statistics and results for both trials including overweight/obese subjects and lean subjects are summarised in the Supplementary Material (Tables S1–S4).

### 3.4. Effect Size and Moderator Variable for Acylated Ghrelin AUC Analysis

**Figure 2:**
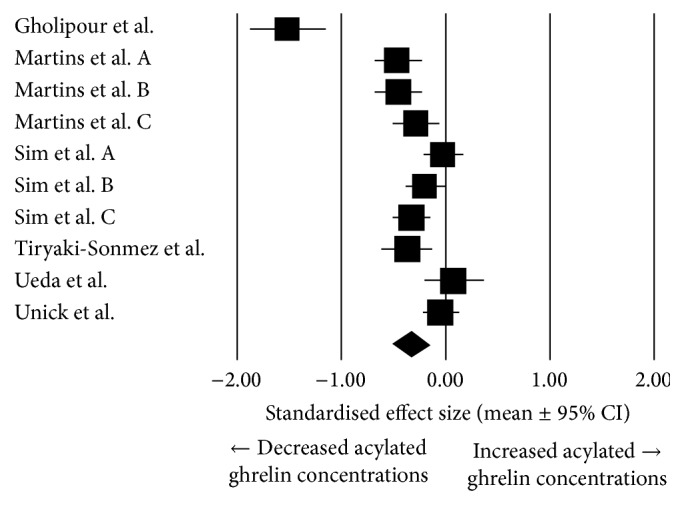
Forest plot of effect sizes (means ± 95% confidence intervals [CIs]) for studies evaluating the influence of acute exercise on acylated ghrelin AUC values in overweight and obese individuals.

In overweight/obese individuals, there was statistically significant moderate suppression in mean acylated ghrelin AUC concentrations in exercise trials compared with resting trials (pooled effect size: −0.34, 95% confidence interval: −0.53 to −0.15; *N* = 10; *P* < 0.001; Figure 2). Heterogeneity was found to be high between these studies (*I*^2^ = 87.7%; *Q* = 73.4, *T*^2^ = 0.084, and *d*_*f*_ = 9). For this reason, a random effects model was chosen to conduct the meta-analyses [27].

Sensitivity analysis showed that the study by Gholipour et al. [20] increased the effect size of exercise on mean acylated ghrelin AUC concentrations. The removal of this study decreased the pooled effect size to −0.23 (95% confidence interval: −0.35 to −0.11, *P* < 0.001).

When data from lean individuals were included into the meta-analysis, with that of the previously reported overweight and obese individuals, the pooled standardised effect size of exercise on acylated ghrelin AUC data was reduced to −0.215 (95% confidence interval: −0.32 to −0.11; *N* = 33; *P* < 0.001).

**Figure 3:**
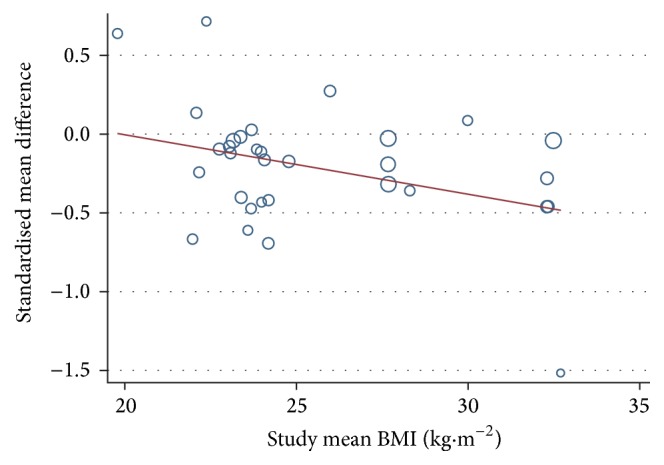
Univariable metaregression for study mean BMI versus the acylated ghrelin AUC values in response to exercise in overweight and obese individuals SMD for acylated ghrelin. Data shown is pooled from the current review and from a previous review [15]. A negative correlation was observed which persisted even when baseline (control) mean ghrelin concentration was added as a covariate.

Using BMI as a moderator in a metaregression model, a higher mean BMI was associated with greater exercise-induced suppression of acylated ghrelin AUC concentration. The slope of regression for BMI was shallow, but significantly negative (95% confidence interval: −0.07 to −0.01; *P* = 0.044; Figure 3). The standardised reduction in acylated ghrelin for exercise versus control conditions was found to be 0.037 units more marked for every 1 kg·m^−2^ increase seen in BMI. Including baseline acylated ghrelin concentrations and BMI into a multiple metaregression model had little effect on the results; the slope of regression became slightly more negative so that the standardised reduction in acylated ghrelin for exercise versus control was 0.040 units more marked for every 1 kg·m^−2^ increase in BMI (95% confidence interval: −0.08 to −0.01; *P* = 0.020). Sensitivity analysis showed that removal of the study by Gholipour et al. [20] decreased the regression slope of the BMI-ghrelin response relationship to −0.02 (95% confidence interval: −0.05 to 0.014; *P* = 0.25). However, no methodological reason was identified for the relatively high effect size in this particular study.

**Figure 4:**
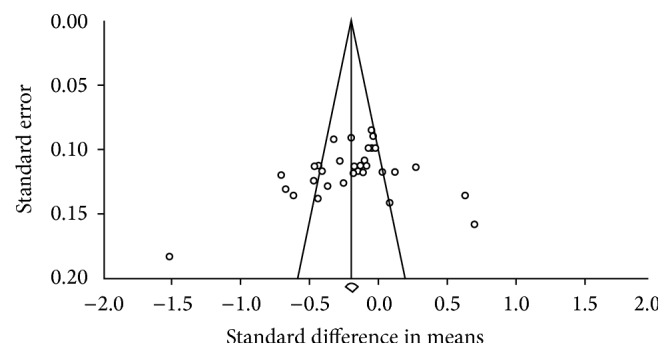
Funnel plot of standard error by standard difference in means for studies evaluating the influence of acute exercise on acylated ghrelin AUC values in overweight/obese individuals.

Inspection of the funnel plot (see Figure 4) and Egger's regression intercept revealed that there was little evidence of small study effects (intercept = −3.647, 95% confidence interval: −9.08 to 1.79, *P* = 0.264).

### 3.5. Effect Size for Total PYY AUC Analysis

**Figure 5:**
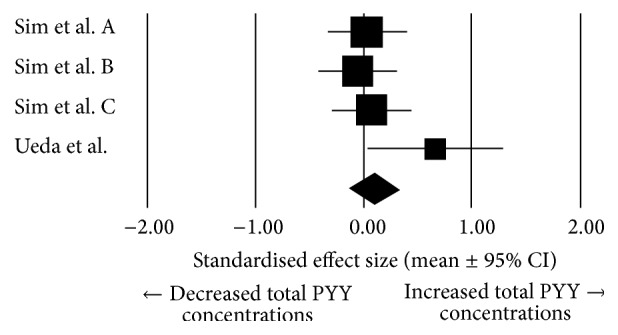
Forest plot of effect sizes (means ± 95% confidence intervals [CIs]) for studies evaluating the influence of acute exercise on total PYY AUC values in overweight and obese individuals.

In overweight/obese individuals, there was a trivial mean effect of exercise on total PYY (pooled effect size: 0.099, 95% confidence interval: −0.13 to 0.31; *N* = 4; Figure 5), and this was not significantly different from zero (*P* = 0.404). Heterogeneity was found to be low between these studies (*I*^2^ = 24.17%; *Q* = 3.96, *T*^2^ = 0.014, and *d*_*f*_ = 3).

### 3.6. Effect Size and Moderator Variable for Total GLP-1 AUC Analysis

**Figure 6:**
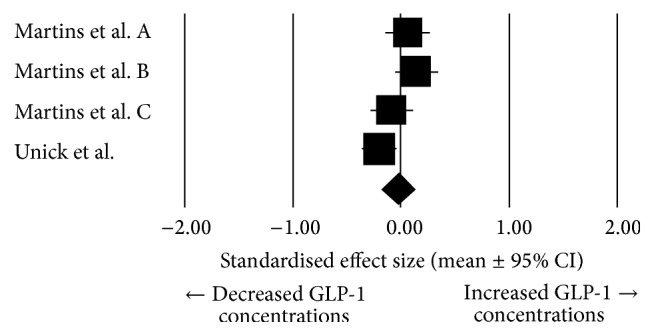
Forest plot of effect sizes (means ± 95% confidence intervals [CIs]) for studies evaluating the influence of acute exercise on total GLP-1 AUC values in overweight and obese individuals.

In overweight/obese individuals, there was a trivial mean effect of exercise on GLP-1 (pooled effect size: −0.026, 95% confidence interval: −0.18 to 0.13; *N* = 4; Figure 6), and this was not significantly different from zero (*P* = 0.749). Heterogeneity was found to be high between these studies (*I*^2^ = 65.7%; *Q* = 8.74, *T*^2^ = 0.017, and *d*_*f*_ = 3).

## 4. Discussion

Understanding the responses of appetite regulatory hormones to exercise and consequently the effect they may have on energy intake and appetite could enhance the understanding of the role of exercise in weight control. The purpose of this review was to examine the concentration changes of acylated ghrelin, total PYY, and total GLP-1 after acute exercise in overweight/obese individuals. We found acylated ghrelin to be moderately suppressed by acute exercise, whilst there were trivial effects of exercise on total PYY and total GLP-1. Ghrelin is an appetite-stimulating hormone [10] and our results suggest that exercise in overweight/obese individuals alters acylated ghrelin in a direction that would be associated with decreased hunger and energy intake. We can only speculate the effects ghrelin has on appetite and food intake as not all studies included these measures in their protocol. Future research should examine energy intake in addition to appetite and appetite regulatory hormone responses to clarify this assumption.

The results of the current review appear to mirror those of lean individuals. In a recent review, lean individuals showed a small reduction in acylated ghrelin after exercise, whilst total PYY and total GLP-1 showed small increases [15]. Our findings suggest that overweight/obese individuals show broadly similar appetite hormone responses to exercise in lean individuals, in such a direction that could alter energy intake and achieve weight loss if sustained over prolonged periods of time. Again, this can only be speculated as the studies included in both the review by Schubert et al. [15] and the current review were acute in nature.

The present metaregression demonstrated greater exercise-induced suppression of acylated ghrelin as BMI increased. Although overweight/obese individuals have shown moderate suppression of acylated ghrelin after exercise, this suppression becomes more prominent as BMI increases from 27.7 to 32.7 kg·m^−2^. This finding differed from that of Schubert et al. [15], where BMI was shown to have no influence on appetite regulatory hormones. Schubert et al. [15] included 23 studies examining responses of lean individuals and two studies with overweight/obese individuals. The inclusion of six, rather than two, studies with overweight/obese individuals in the present metaregression may explain the differences found between the two reviews.

The current review found overweight/obese individuals to express a moderate reduction in acylated ghrelin during exercising conditions. Large variations in fasting and postprandial ghrelin concentrations between individuals make it difficult to establish the clinical relevance that exercise has on this hormone. In lean individuals, circulating concentrations of acylated ghrelin in the range of 40–67 pg/mL may be expected in fasting conditions [28], with obese individuals expressing lower concentrations [25, 29]. We attempted to control for differences in baseline levels of acylated ghrelin between studies by accounting for fasting values observed in resting trials. This had negligible influence on the relationship between increased BMI and acylated ghrelin.

The current review found three studies that examined the acute effects of exercise on appetite regulatory hormones in males, two studies in females, and one study including both males and females. Due to the limited number of studies in this review, no conclusions can be drawn upon the effect of exercise and sex on appetite regulatory hormones. It has been hypothesised that exercise could influence acylated ghrelin differently in obese males and females. In one study, after four days of consecutive exercise, females experienced an increase in acylated ghrelin, whereas males showed no change [30]. This suggests that females may be prone to increasing energy intake after exercise training. However, after acute exercise, lean individuals have shown no sex difference in responses of PYY_3-36_ or acylated ghrelin [31]. Similar relative energy intakes were observed in males and females, suggesting that acute exercise is equally effective for both sexes. Future research is required to understand and compare the responses of males and females.

The current review has several limitations. First, only six studies were identified as relevant following our literature searches. We recognise that meta-analyses are not immune from statistical power-related issues and that the pooling of data from such a small number of studies may still provide relatively low statistical precision (wide confidence interval for pooled effect). For example, despite finding substantial heterogeneity amongst studies in which ghrelin and GLP-1 were measured, precise analyses such as one for the presence of outliers could not be performed due to the small number of studies. We restricted our search to acute exercise trials; further reviews should examine the effects of repeated bouts of exercise and exercise training on appetite regulatory hormones in overweight/obese individuals although at present the literature on this aspect is very limited. The longest trial length in the current review was 3 h; future research should examine what happens to this population later on in the exercising day. Despite trials only lasting 2-3 h, protocols differed widely between studies, potentially confounding appetite regulatory AUC estimations, specifically the timing of exercise and meal provision within the study protocol. AUC calculations were made over the duration of each trial, irrespective of when exercise occurred. The inclusion of rest periods prior to exercise into AUC calculations could potentially underestimate the effect of exercise on hormone responses. Additionally, studies differed by exercising participants in both fasting and fed states. Further still, studies varied in meal provision (standardised, ad libitum, or no meal) after exercise. Together, these could further cofound the study of hormonal responses to exercise. We metaregressed the SMD in acylated ghrelin versus study mean BMI. Such study-level explorations of potential moderators of effect size should be interpreted with caution as within-study relationships can sometimes disagree with between-study relationships [32]. The average BMI of the participants in this review was 30.6 kg·m^−2^ (i.e., borderline obese); therefore, we cannot generalise the findings of this review to those who fall higher into the obese or severely obese category. Despite our best efforts, we cannot guarantee that we captured all the relevant studies for this review. The fact that some researchers studied multiple exercise conditions in their experiments raises the issue of “double counting” in meta-analyses [33]. We acknowledge that our estimates of variance may have been affected by this issue. Nevertheless, it was extremely difficult to eradicate this problem because all studies were crossover in design (rather than separate study arms) and we already had to impute a within-subjects correlation for the meta-analysis (because study authors tend not to report directly the standard deviation of the mean change). Finally, we cannot directly link the effects of exercise on appetite regulatory hormones to weight loss and management due to the acute nature of the studies and the lack of consistency in studies including energy intake and appetite ratings.

## 5. Conclusions

An evidence synthesis of the six studies on overweight/obese individuals indicated that a moderate reduction in acylated ghrelin occurs after acute exercise. Only trivial effects of exercise were quantified for total PYY and GLP-1.

## Supplementary Material

The supplementary material contains details of the search strategy used for the current literature search. Tables S1 to S4 summarise individual study statistics and results for control and exercise trials for overweight/obese and lean subjects identified for the current and previous reviews [15].

## Figures and Tables

**Table 1:** Hormone area-under-the-curve (AUC) data for the six studies included in the meta-analysis.

Study	Participants		Intervention	Hormone AUC (pg·mL^−1^)		
				Acylated ghrelin	Total PYY	Total GLP-1
	*N*	BMI (kg·m^−2^)				
Gholipour et al. [20]	9 (males)	32.7 ± 0.8	36 min treadmill run: 10 min, 10 min, 5 min, and 2 min at 65% $\dot{\text{V}}{\text{O}}_{\text{2}\max}$, separated by 3 min at 3 km·h^−1^	CON: 3512 ± 654 EX: 1935 ± 302^*∗*^	NM	NM

Martins et al. [21]	12 (7 females and 5 males)	32.3 ± 2.7	Cycling at 85–90% HR_max_ until 250 kcal is expended: 8 s all-out sprinting separated by 12 s easy pedalling (average duration: 18 ± 3 min)	CON: 3921 ± 1318 EX: 3315 ± 1219^*∗*^	NM	CON: 4181 ± 1262 EX: 4272 ± 969
			Cycling at 70% HR_max_ until 250 kcal is expended: continuous cycling (average duration: 27 ± 6 min)	CON: 3921 ± 1318 EX: 3296 ± 1058^*∗*^	NM	CON: 4181 ± 1262 EX: 4638 ± 1305
			Cycling at 85–90% HR_max_ until 125 kcal is expended: 8 s all-out sprinting separated by 12 s easy pedalling (average duration: 9 ± 2 min)	CON: 3921 ± 1318 EX: 3532 ± 1393	NM	CON: 4181 ± 1262 EX: 4072 ± 1156

Sim et al. [22]	17 (males)	27.7 ± 1.6	30 min continuous cycling at 60% $\dot{\text{V}}{\text{O}}_{\text{2peak}}$	CON: 70 ± 37 EX: 69 ± 30	CON: 85 ± 43 EX: 87 ± 37	NM
			30 min cycling: alternating between 60 s at 100% $\dot{\text{V}}O_{2peak}$ and 240 s at 50% $\dot{\text{V}}{\text{O}}_{\text{2peak}}$	CON: 70 ± 37 EX: 62 ± 28	CON: 85 ± 43 EX: 83 ± 40	
			30 min cycling: alternating between 15 s at 170% $\dot{\text{V}}O_{2peak}$ and 60 s at 32% $\dot{\text{V}}{\text{O}}_{\text{2peak}}$	CON: 70 ± 37 EX: 56 ± 26	CON: 85 ± 43 EX: 88 ± 34	

Tiryaki-Sonmez et al. [23]	9 (females)	28.3 ± 1.8	60 min running at 53% $\dot{\text{V}}{\text{O}}_{\text{2max}}$	CON: 51 ± 8 EX: 47 ± 5	NM	NM

Ueda et al. [13]	7 (males)	30.0 ± 3.1	60 min cycling at 50% $\dot{\text{V}}{\text{O}}_{\text{2max}}$	CON: 15779 ± 10046 EX: 16641 ± 10725	CON: 393 ± 50^*∗*^ EX: 425 ± 46^*∗*^	NM

Unick et al. [24]	19 (females)	32.5 ± 4.3	Walking at 70–75% age predicted HR_max_ until 3.0 kcal·kg^−1^ of body weight is expended (average energy expenditure: 354 ± 72 kcal; average duration: 42 ± 8 min)	CON: 6527 ± 2646 EX: 6361 ± 3339	NM	CON: 211400 ± 51600 EX: 201200 ± 49400^*∗*^

BMI: body mass index; CON: resting control trial; EX: exercise trial; GLP-1: glucagon-like peptide-1; HR_max_: maximum heart rate; NA: AUC data not available; NM: not measured; PYY: peptide-YY; $\dot{\text{V}}{\text{O}}_{\text{2max}}$: maximum oxygen uptake.

^*∗*^Significantly different from control (*P* < 0.05). Area-under-the-curve values were calculated using hours as the unit of time in some studies and minutes as the unit of time in others.

## References

[1] World Health Organisation (2014). *Obesity and Overweight*.

[2] National Health Service (2014). *Obesity*.

[3] World Health Organisation (2010). *Global Recommendations on Physical Activity for Health*.

[4] King N. A., Burley V. J., Blundell J. E. (1994). Exercise-induced suppression of appetite: effects on food intake and implications for energy balance. *European Journal of Clinical Nutrition*.

[5] Broom D. R., Batterham R. L., King J. A., Stensel D. J. (2009). Influence of resistance and aerobic exercise on hunger, circulating levels of acylated ghrelin, and peptide YY in healthy males. *American Journal of Physiology—Regulatory Integrative and Comparative Physiology*.

[6] Broom D. R., Stensel D. J., Bishop N. C., Burns S. F., Miyashita M. (2007). Exercise-induced suppression of acylated ghrelin in humans. *Journal of Applied Physiology*.

[7] King J. A., Miyashita M., Wasse L. K., Stensel D. J. (2010). Influence of prolonged treadmill running on appetite, energy intake and circulating concentrations of acylated ghrelin. *Appetite*.

[8] King J. A., Wasse L. K., Broom D. R., Stensel D. J. (2010). Influence of brisk walking on appetite, energy intake, and plasma acylated ghrelin. *Medicine and Science in Sports and Exercise*.

[9] King J. A., Wasse L. K., Ewens J. (2011). Differential acylated ghrelin, peptide YY3-36, appetite, and food intake responses to equivalent energy deficits created by exercise and food restriction. *The Journal of Clinical Endocrinology & Metabolism*.

[10] Cummings D. E., Purnell J. Q., Frayo R. S., Schmidova K., Wisse B. E., Weigle D. S. (2001). A preprandial rise in plasma ghrelin levels suggests a role in meal initiation in humans. *Diabetes*.

[11] Harrold J. A., Dovey T. M., Blundell J. E., Halford J. C. G. (2012). CNS regulation of appetite. *Neuropharmacology*.

[12] Karra E., Chandarana K., Batterham R. L. (2009). The role of peptide YY in appetite regulation and obesity. *The Journal of Physiology*.

[13] Ueda S.-Y., Yoshikawa T., Katsura Y., Usui T., Nakao H., Fujimoto S. (2009). Changes in gut hormone levels and negative energy balance during aerobic exercise in obese young males. *Journal of Endocrinology*.

[14] Schubert M. M., Desbrow B., Sabapathy S., Leveritt M. (2013). Acute exercise and subsequent energy intake. A meta-analysis. *Appetite*.

[15] Schubert M. M., Sabapathy S., Leveritt M., Desbrow B. (2014). Acute exercise and hormones related to appetite regulation: a meta-analysis. *Sports Medicine*.

[16] Deeks J. J., Higgins J. P. T., Altman D. G., Higgins J. P. T., Green S. (2011). Chapter 9: analysing data and undertaking meta-analyses. *Cochrane Handbook for Systematic Reviews of Interventions Version 5.1.0*.

[17] Higgins J. P. T., Deeks J. J., Higgins J. P. T., Green S. (2011). Selecting studies and collecting data. *Cochrane Handbook for Systematic Reviews of Interventions Version 5.1.0*.

[18] Hopkins W. G., Batterham A. M. (2016). Error rates, decisive outcomes and publication bias with several inferential methods. *Sports Medicine*.

[19] Hopkins W. G., Marshall S. W., Batterham A. M., Hanin J. (2009). Progressive statistics for studies in sports medicine and exercise science. *Medicine & Science in Sports & Exercise*.

[20] Gholipour M., Kordi M., Taghikhani M., Ravasi A., Gaeini A., Tabrizi A. (2011). The acute effects of intermittent treadmill running on hunger and plasma acylated ghrelin concentration in individuals with obesity. *Tehran University Medical Journal*.

[21] Martins C., Stensvold D., Finlayson G. (2015). Effect of moderate- and high-intensity acute exercise on appetite in obese individuals. *Medicine and Science in Sports and Exercise*.

[22] Sim A. Y., Wallman K. E., Fairchild T. J., Guelfi K. J. (2014). High-intensity intermittent exercise attenuates ad-libitum energy intake. *International Journal of Obesity*.

[23] Tiryaki-Sonmez G., Ozen S., Bugdayci G. (2013). Effect of exercise on appetite-regulating hormones in overweight women. *Biology of Sport*.

[24] Unick J. L., Otto A. D., Goodpaster B. H., Helsel D. L., Pellegrini C. A., Jakicic J. M. (2010). Acute effect of walking on energy intake in overweight/obese women. *Appetite*.

[25] Shiiya T., Nakazato M., Mizuta M. (2002). Plasma ghrelin levels in lean and obese humans and the effect of glucose on ghrelin secretion. *Journal of Clinical Endocrinology and Metabolism*.

[26] Sterne J. A. C., Egger M., Moher D., Higgins J. P. T., Green S. (2011). Addressing reporting biases. *Cochrane Handbook for Systematic Reviews of Interventions Version 5.1.0 (updated March 2011)*.

[27] Ades A. E., Lu G., Higgins J. P. T. (2005). The interpretation of random-effects meta-analysis in decision models. *Medical Decision Making*.

[28] Sato T., Nakamura Y., Shiimura Y., Ohgusu H., Kangawa K., Kojima M. (2012). Structure, regulation and function of ghrelin. *The Journal of Biochemistry*.

[29] Tschöp M., Weyer C., Tataranni P. A., Devanarayan V., Ravussin E., Heiman M. L. (2001). Circulating ghrelin levels are decreased in human obesity. *Diabetes*.

[30] Hagobian T. A., Sharoff C. G., Stephens B. R. (2009). Effects of exercise on energy-regulating hormones and appetite in men and women. *American Journal of Physiology—Regulatory Integrative and Comparative Physiology*.

[31] Hagobian T. A., Yamashiro M., Hinkel-Lipsker J., Streder K., Evero N., Hackney T. (2013). Effects of acute exercise on appetite hormones and ad libitum energy intake in men and women. *Applied Physiology, Nutrition and Metabolism*.

[32] Petkova E., Tarpey T., Huang L., Deng L. (2013). Interpreting meta-regression: application to recent controversies in antidepressants' efficacy. *Statistics in Medicine*.

[33] Senn S. J. (2009). Overstating the evidence—double counting in meta-analysis and related problems. *BMC Medical Research Methodology*.

