# Supplementary material for: Acute Exercise and Appetite-Regulating Hormones in Overweight and Obese Individuals: A Meta-Analysis
Source: J Obes. 2016 Dec 27;2016:2643625. doi: 10.1155/2016/2643625 (PMC5223036; doi:10.1155/2016/2643625)
Supplement: Supplementary file 1 — The supplementary material contains details of the search strategy used for the current literature search. Tables S1 to S4 summarise individual study statistics and results for control and exercise trials for overweight/obese and lean subjects identified for the current and previous reviews [15]. [file 2643625.f1.pdf]

Supplementary material

Search strategy

Keywords

Study Population	*Intervention*
Obese	Exercise; Physical exercise; Aerobic exercise
Overweight	Physical activity
	Energy expenditure
	Energy intake; Caloric intake
	Food intake
	Appetite
	Hunger
	Appetite hormones; Appetite regulating hormones
	Ghrelin; Acylated ghrelin; Total ghrelin; Acyl ghrelin
	Peptide YY; PYY; Peptide YY 3-36; PYY3-36; PYY (3-36); Total PYY
	Glucagon like peptide 1; Glucagon-like peptide-1; GLP-1; total GLP-1; active GLP-1; GLP-1 (7-36); GLP-1 (9-36)

I. Obese or overweight
II. Exercise or physical exercise or aerobic exercise or physical activity or energy expenditure
III. Energy intake or caloric intake or food intake
IV. Appetite regulatory hormones or appetite hormones
V. Peptide YY or PYY or Peptide YY 3-36 or PYY3-36 or PYY (3-36) or Total PYY
VI. Ghrelin or Acylated ghrelin or Total ghrelin or Acyl ghrelin
VII. Glucagon like peptide 1 or Glucagon-like peptide-1 or GLP-1 or total GLP-1 or active GLP-1 or GLP-1 (7-36) or GLP-1 (9-36)
VIII. Obese or overweight and Exercise or physical exercise or aerobic exercise or physical activity or energy expenditure (I and II)
IX. Obese or overweight and Energy intake or caloric intake or food intake (I and III)
X. Obese or overweight and Appetite regulatory hormones or appetite hormones (I and IV)
XI. Obese or overweight and Peptide YY or PYY or Peptide YY 3-36 or PYY3-36 or PYY (3-36) or Total PYY (I and V)
XII. Obese or overweight and Ghrelin or Acylated ghrelin or Total ghrelin or Acyl ghrelin (I and VI)
XIII. Obese or overweight and Glucagon like peptide 1 or Glucagon-like peptide-1 or GLP-1 or total GLP-1 or active GLP-1 or GLP-1 (7-36) or GLP-1 (9-36) (I and VII)
XIV. Obese or overweight and Exercise or physical exercise or aerobic exercise or physical activity or energy expenditure and Energy intake or caloric intake or food intake (I and II and III)
XV. Obese or overweight and Exercise or physical exercise or aerobic exercise or physical activity or energy expenditure and Appetite regulatory hormones or appetite hormones (I and II and IV)
XVI. Obese or overweight and Exercise or physical exercise or aerobic exercise or physical activity or energy expenditure and Peptide YY or PYY or Peptide YY 3-36 or PYY3-36 or PYY (3-36) or Total PYY (I and II and V)
XVII. Obese or overweight and Exercise or physical exercise or aerobic exercise or physical activity or energy expenditure and Ghrelin or Acylated ghrelin or Total ghrelin or Acyl ghrelin (I and II and VI)
XVIII. Obese or overweight and Exercise or physical exercise or aerobic exercise or physical activity or energy expenditure and Glucagon like peptide 1 or Glucagon-like peptide-1 or GLP-1 or total GLP-1 or active GLP-1 or GLP-1 (7-36) or GLP-1 (9-36) (I and II and VII)

XIX. Obese or overweight (RCT filter)

XX. Exercise or physical exercise or aerobic exercise or physical activity or energy expenditure (RCT filter)

XXI. Energy intake or caloric intake or food intake (RCT filter)

XXII. Appetite regulatory hormones or appetite hormones (RCT filter)

XXIII. Peptide YY or PYY or Peptide YY 3-36 or PYY3-36 or PYY (3-36) or Total PYY (RCT filter)

XXIV. Ghrelin or Acylated ghrelin or Total ghrelin or Acyl ghrelin (RCT filter)

XXV. Glucagon like peptide 1 or Glucagon-like peptide-1 or GLP-1 or total GLP-1 or active GLP-1 or GLP-1 (7-36) or GLP-1 (9-36) (RCT filter)

XXVI. Obese or overweight and Exercise or physical exercise or aerobic exercise or physical activity or energy expenditure (I and II) (RCT filter)

XXVII. Obese or overweight and Energy intake or caloric intake or food intake (I and III) (RCT filter)

XXVIII. Obese or overweight and Appetite regulatory hormones or appetite hormones (I and IV) (RCT filter)

XXIX. Obese or overweight and Peptide YY or PYY or Peptide YY 3-36 or PYY3-36 or PYY (3-36) or Total PYY (I and V) (RCT filter)

XXX. Obese or overweight and Ghrelin or Acylated ghrelin or Total ghrelin or Acyl ghrelin (I and VI) (RCT filter)

XXXI. Obese or overweight and Glucagon like peptide 1 or Glucagon-like peptide-1 or GLP-1 or total GLP-1 or active GLP-1 or GLP-1 (7-36) or GLP-1 (9-36) (I and VII) (RCT filter)

XXXII. Obese or overweight and Exercise or physical exercise or aerobic exercise or physical activity or energy expenditure and Energy intake or caloric intake or food intake (I and II and III) (RCT filter)

XXXIII. Obese or overweight and Exercise or physical exercise or aerobic exercise or physical activity or energy expenditure and Appetite regulatory hormones or appetite hormones (I and II and IV) (RCT filter)

XXXIV. Obese or overweight and Exercise or physical exercise or aerobic exercise or physical activity or energy expenditure and Peptide YY or PYY or Peptide YY 3-36 or PYY3-36 or PYY (3-36) or Total PYY (I and II and V) (RCT filter)

XXXV. Obese or overweight and Exercise or physical exercise or aerobic exercise or physical activity or energy expenditure and Ghrelin or Acylated ghrelin or Total ghrelin or Acyl ghrelin (I and II and VI) (RCT filter)

XXXVI. Obese or overweight and Exercise or physical exercise or aerobic exercise or physical activity or energy expenditure and Glucagon like peptide 1 or Glucagon-like peptide-1 or GLP-1 or total GLP-1 or active GLP-1 or GLP-1 (7-36) or GLP-1 (9-36) (I and II and VII) (RCT filter)

Table S1. Individual study characteristics for studies evaluating acylated ghrelin

Study	Category	Standard difference in means	Standard error	Variance	Lower 95% confidence interval	Upper 95% confidence interval	Z- value	p-value	Weight (%)
Gholipour, M.	Overweight/obese	-1.517	0.183	0.033	-1.876	-1.159	-8.295	0.000	8.303
Martins, S. A	Overweight/obese	-0.468	0.114	0.013	-0.691	-0.245	-4.115	0.000	10.064
Martins, S. B	Overweight/obese	-0.456	0.113	0.013	-0.678	-0.234	-4.019	0.000	10.070
Martins, S. C	Overweight/obese	-0.284	0.110	0.012	-0.500	-0.069	-2.582	0.010	10.148
Sim, A. A	Overweight/obese	-0.032	0.091	0.008	-0.210	0.146	-0.353	0.724	10.578
Sim, A. B	Overweight/obese	-0.201	0.092	0.008	-0.381	-0.021	-2.193	0.028	10.559
Sim, A. C	Overweight/obese	-0.325	0.093	0.009	-0.507	-0.142	-3.490	0.000	10.529
Tiryaki-Sonmez, G.	Overweight/obese	-0.367	0.129	0.017	-0.619	-0.114	-2.847	0.004	9.697
Ueda, S.-y.	Overweight/obese	0.082	0.142	0.020	-0.196	0.359	0.577	0.564	9.374
Unick, J.L.	Overweight/obese	-0.047	0.086	0.007	-0.216	0.121	-0.552	0.581	10.678
Mean		-0.340	0.099	0.010	-0.533	-0.146	-3.443	0.001	100
Broom et al. 2007	Lean	-0.253	0.127	0.016	-0.501	-0.004	-1.994	0.046	4.290
Broom et al. 2009	Lean	-0.085	0.113	0.013	-0.307	0.136	-0.753	0.451	4.427
Broom et al. 2009-1	Lean	-0.125	0.113	0.013	-0.347	0.097	-1.104	0.269	4.424
Shorten et al. 2009	Lean	-0.172	0.114	0.013	-0.395	0.051	-1.512	0.131	4.421
Ueda et al. 2009a	Lean	0.701	0.158	0.025	0.391	1.010	4.439	0.000	3.960
King et al. 2010b	Lean	-0.620	0.136	0.019	-0.887	-0.353	-4.553	0.000	4.192
King et al. 2010a	Lean	-0.023	0.100	0.010	-0.219	0.173	-0.233	0.816	4.549
Balaguera-Cortes et al. 2011	Lean	0.023	0.118	0.014	-0.209	0.255	0.197	0.844	4.374

Balaguera-Cortes et al. 2011-1	Lean	-0.476	0.125	0.016	-0.721	-0.231	-3.813	0.000	4.309
King et al. 2011a	Lean	-0.100	0.108	0.012	-0.312	0.112	-0.924	0.355	4.472
King et al. 2011b	Lean	-0.047	0.100	0.010	-0.243	0.149	-0.468	0.640	4.549
Vatansever-Ozen, 2011	Lean	-0.672	0.131	0.017	-0.929	-0.415	-5.129	0.000	4.246
Becker et al. 2012	Lean	-0.437	0.138	0.019	-0.708	-0.165	-3.155	0.002	4.168
Kelly et al. 2012	Lean	-0.106	0.119	0.014	-0.339	0.126	-0.896	0.370	4.371
Larson Meyer et al. 2012	Lean	0.631	0.137	0.019	0.363	0.899	4.621	0.000	4.187
Larson Meyer et al. 2012a	Lean	0.123	0.119	0.014	-0.110	0.356	1.036	0.300	4.370
Wasse et al. 2012	Lean	-0.181	0.119	0.014	-0.415	0.053	-1.515	0.130	4.365
Wasse et al. 2013-1	Lean	-0.407	0.117	0.014	-0.637	-0.176	-3.463	0.001	4.384
Wasse et al. 2013	Lean	-0.412	0.117	0.014	-0.642	-0.181	-3.502	0.000	4.383
Deighton et al. 2013	Lean	-0.427	0.113	0.013	-0.648	-0.206	-3.785	0.000	4.428
Deighton et al. 2013-1	Lean	-0.700	0.121	0.015	-0.936	-0.464	-5.808	0.000	4.353
Hagobian et al. 2013	Lean	0.270	0.115	0.013	0.044	0.495	2.347	0.019	4.409
Hagobian et al. 2013-1	Lean	-0.119	0.119	0.014	-0.352	0.113	-1.004	0.315	4.370
Mean		-0.159	0.067	0.005	-0.291	-0.027	-2.358	0.018	100

Table S2. Individual study characteristics for studies evaluating total PYY

Study	Category	Standard difference in means	Standard error	Variance	Lower 95% confidence interval	Upper 95% confidence interval	Z- value	p-value	Weight (%)
Sim, A. A	Overweight/obese	0.034	0.185	0.034	-0.328	0.396	0.186	0.853	29.296
Sim, A. B	Overweight/obese	-0.052	0.185	0.034	-0.414	0.310	-0.281	0.779	29.280
Sim, A. C	Overweight/obese	0.078	0.185	0.034	-0.285	0.441	0.422	0.673	29.245
Ueda, S.-y.	Overweight/obese	0.666	0.318	0.101	0.042	1.289	2.092	0.036	12.179
Mean		0.099	0.118	0.014	-0.133	0.331	0.835	0.404	100
Martins et al. 2007	Lean	0.056	0.311	0.097	-0.553	0.666	0.181	0.856	4.857
Broom et al. 2009a	Lean	0.647	0.253	0.064	0.152	1.142	2.564	0.010	7.373
Broom et al. 2009b	Lean	-0.086	0.230	0.053	-0.537	0.365	-0.375	0.707	8.885
Shorten et al. 2009	Lean	0.165	0.231	0.053	-0.288	0.619	0.716	0.474	8.798
Ueda et al. 2009a	Lean	0.465	0.303	0.092	-0.129	1.058	1.533	0.125	5.122
Balaguera-Cortes et al. 2011a	Lean	0.085	0.241	0.058	-0.388	0.558	0.354	0.723	8.078
Balaguera-Cortes et al. 2011b	Lean	-0.133	0.242	0.059	-0.607	0.341	-0.549	0.583	8.037
Kelly et al. 2012	Lean	0.153	0.242	0.059	-0.321	0.628	0.633	0.527	8.013
Larson Meyer et al. 2012a	Lean	0.023	0.254	0.064	-0.474	0.521	0.093	0.926	7.295
Larson Meyer et al. 2012b	Lean	0.553	0.259	0.067	0.046	1.059	2.137	0.033	7.034
Wasse et al. 2013	Lean	0.236	0.244	0.060	-0.242	0.715	0.968	0.333	7.888
Deighton et al. 2012a	Lean	0.337	0.226	0.051	-0.106	0.780	1.493	0.135	9.205
Deighton et al. 2012b	Lean	0.259	0.223	0.050	-0.179	0.697	1.158	0.247	9.414
Mean		0.204	0.069	0.005	0.070	0.339	2.979	0.003	100

Table S3. Individual study characteristics for studies evaluating total GLP-1

Study	Category	Standard difference in means	Standard error	Variance	Lower 95% confidence interval	Upper 95% confidence interval	Z- value	p-value	Weight (%)
Martins, S. A	Overweight/obese	0.066	0.100	0.010	-0.131	0.262	0.655	0.512	24.108
Martins, S. B	Overweight/obese	0.146	0.101	0.010	-0.051	0.343	1.450	0.147	24.033
Martins, S. C	Overweight/obese	-0.087	0.100	0.010	-0.284	0.109	-0.872	0.383	24.093
Unick, J.L.	Overweight/obese	-0.200	0.080	0.006	-0.358	-0.043	-2.497	0.013	27.765
Mean									100
Martins et al. 2007	Lean	0.628	0.155	0.024	0.325	0.932	4.060	0.000	13.908
Ueda et al. 2009a	Lean	0.381	0.136	0.018	0.116	0.647	2.813	0.005	15.608
Ueda et al. 2009b	Lean	0.518	0.117	0.014	0.290	0.747	4.442	0.000	17.441
Ueda et al. 2009b-1	Lean	0.518	0.117	0.014	0.290	0.747	4.443	0.000	17.441
Larson Meyer et al. 2012	Lean	0.155	0.116	0.013	-0.072	0.383	1.336	0.182	17.492
Larson Meyer et al. 2012a	Lean	0.136	0.110	0.012	-0.079	0.352	1.239	0.215	18.109
Mean									100

Table S4. Individual study characteristics for studies evaluating insulin

Study	Category	Standard difference in means	Standard error	Variance	Lower 95% confidence interval	Upper 95% confidence interval	Z- value	p-value	Weight (%)
Martins, S. A	Overweight/obese	-0.698	0.241	0.058	-1.171	-0.226	-2.899	0.004	13.517
Martins, S. B	Overweight/obese	-0.275	0.220	0.048	-0.706	0.156	-1.249	0.212	13.986
Martins, S. C	Overweight/obese	-0.087	0.216	0.047	-0.512	0.337	-0.404	0.686	14.066
Sim, A. A	Overweight/obese	0.384	0.188	0.035	0.015	0.752	2.040	0.041	14.677
Sim, A. B	Overweight/obese	0.226	0.184	0.034	-0.134	0.586	1.230	0.219	14.766
Sim, A. C	Overweight/obese	0.352	0.187	0.035	-0.014	0.719	1.883	0.060	14.698
Ueda, S-y.	Overweight/obese	-0.942	0.206	0.043	-1.346	-0.537	-4.566	0.000	14.289
Mean		-0.138	0.197	0.039	-0.524	0.248	-0.702	0.483	100
